# The metastasis landscape of *Clonorchis sinensis*-associated hepatocellular carcinoma: an integrated multi-omics and clinical study

**DOI:** 10.3389/fimmu.2026.1723156

**Published:** 2026-01-28

**Authors:** Lingling Zhou, Lin Sun, Xuhang Huang, Junxian Chen, Taijun Huang, Yulong Xu, Xiaorong Luo, Caibiao Wei, Fengfei Liu, Xiaolan Pan, Madanni Dong, Jingyu Su, Weilong Yang, Min Fang

**Affiliations:** 1 Department of Clinical Laboratory, Guangxi Medical University Cancer Hospital, Nanning, China; 2 Department of Clinical Laboratory, the First Affiliated Hospital of Guangxi Medical University, Nanning, China; 3 Department of Blood Transfusion Room, Guangxi Medical University cancer hospital, Nanning, China; 4 Department of Bioinformatics, The Province and Ministry Co-sponsored Collaborative Innovation Center for Medical Epigenetics, Key Laboratory of Immune Microenvironment and Disease (Ministry of Education), School of Basic Medical Sciences, Tianjin Medical University, Tianjin, China; 5 Genetic Metabolism Center laboratory, Guangxi Zhuang Autonomous Region Maternal and Child Health Care Hospital, Nanning, China; 6 Guangzhou Women and Children’s Medical Center, Guangzhou Medical University, Guangzhou, China; 7 Engineering Research Center for Tissue & Organ Injury and Repair Medicine, Guangxi Medical University Cancer Hospital, Nanning, China

**Keywords:** clinical study, *Clonorchis sinensis*, hepatocellular carcinoma, metastasis, multi-omics

## Abstract

**Background:**

Hepatocellular carcinoma (HCC) patients with *Clonorchis sinensis* (*Cs*) infection tend to exhibit a poorer prognosis compared to those without infection. Nevertheless, the molecular mechanisms underlying *Cs*-associated HCC, particularly those linked to metastatic progression, remain poorly understood. This study therefore seeks to elucidate the role of *C. sinensis* infection in promoting metastasis.

**Methods:**

Through a clinical retrospective analysis, we compared overall survival and metastasis incidence between HCC patients with and without *Cs* infection. To explore the underlying mechanisms, we conducted integrated multi-omics analyses—including RNA-seq, miRNA-seq, ATAC-seq, WGBS-seq, oxWGBS-seq, and ChIP-seq—to profile 369 metastasis-related genes in *Cs*^+^ and *Cs*^-^ HCC tumors. The expression of three key metastasis-related genes was further validated by RT–qPCR, and Transwell and wound-healing assays were performed *in vitro* to confirm the pro-metastatic effect of *Cs* infection on HCC cells.

**Results:**

In HCC patients, *Cs* infection was associated with poorer overall survival and an increased metastasis rate. We identified 20 metastasis-related genes, with *SPP1*, *MMP2*, and *VCAM1* as central hubs, together with 41 interacting miRNAs and 71 accessible promoter regions. Histone modifications—particularly H3K9ac, H3K27ac and H3K4me3—were correlated with chromatin accessibility in the promoters of these genes. Molecular experiments further demonstrated that *Cs* infection enhances the metastatic potential of HCC.

**Conclusions:**

Our study reveals that *Cs* infection promotes HCC metastasis through gene and epigenetic alterations, providing mechanistic insights and identifying potential targets for early intervention.

## 1 Introduction

Liver cancer is one of the most common malignant tumors worldwide and the third leading cause of cancer-related deaths, with a five-year survival rate of only 22% (1–3). Among these, hepatocellular carcinoma (HCC) accounts for approximately 80%–90% of liver cancers (4). Due to the absence of typical early symptoms, HCC patients are often diagnosed at an advanced stage, frequently with multiple metastatic lesions (5). The five-year survival rate for patients with distant metastases is approximately 17% (6). Metastasis is therefore a critical determinant of poor prognosis and therapeutic difficulty in HCC. As a highly invasive malignancy, HCC not only tends to form multiple foci within the liver but also metastasizes extrahepatically via hematogenous, lymphatic, and direct implantation pathways. The lungs and bones are common sites of metastasis, accounting for up to 60% and 40% of cases, respectively (7). HCC metastasis involves multiple molecular mechanisms, including epithelial-mesenchymal transition (EMT), tumor angiogenesis, immune evasion, and extracellular matrix degradation mediated by matrix metalloproteinases (MMPs) (8–13). These processes collectively drive HCC cells to breach the basement membrane, invade the circulation, and establish distant metastases. Elucidating the molecular mechanisms and risk factors underlying HCC metastasis is therefore essential for early detection, targeted intervention, and improved prognosis.

*Clonorchis sinensis* (*Cs*), also known as the liver fluke, is a significant foodborne parasite primarily endemic to Southeast Asian regions including China, Japan, South Korea, and Vietnam, with approximately 35 million people infected globally (14, 15). *Cs* primarily parasitizes the bile ducts and gallbladder, infecting humans through the consumption of undercooked freshwater fish containing metacercariae (14, 16). Long-term infection can cause chronic cholangitis, cholestasis, cholangiolar epithelial hyperplasia, and liver fibrosis (16, 17). It is classified as a Group 1 carcinogen by the International Agency for Research on Cancer (IARC) (18). In regions with high prevalence of *Cs*, infection has been observed to correlate with increased incidence and metastatic potential of HCC (19, 20). Recent studies have shown that *Cs* secretory products upregulate MMP-2 and MMP-9, while *Cs* granule proteins (*Cs*GRN) activate the EGFR–PI3K/AKT signaling pathway, accelerating extracellular matrix ECM degradation and remodeling (21–23). Concurrently, *Cs*-induced chronic inflammation and fibrosis involve VEGF/Ang signaling activation, promoting neovascularization (24). Furthermore, in *Cs*-infected HCC patients, cancer stem cell (CSC) markers CK19 and EpCAM exhibit significantly elevated expression, which are recognized as key drivers of HCC heterogeneity, drug resistance, and metastasis (25–27). These findings link *Cs* infection to the metastatic clinical features of HCC and are associated with poorer overall survival outcomes in patients (25).

However, although epidemiological and experimental evidence suggests that *Cs* infection may be associated with an increased risk of HCC metastasis (24, 25), robust evidence supporting its independent and direct role in HCC progression remains lacking. Observational studies are often confounded by underlying disease-related factors, and most existing data are derived from early associative studies or animal models, the causal relationship and direct mechanisms linking *Cs* infection to enhanced HCC metastasis remain to be elucidated (28, 29). Therefore, to investigate the role of *Cs* infection in HCC metastasis, we integrated multi-omics analyses of *Cs*-infected tumor tissues to assess the effects of *Cs* infection on metastasis-related gene expression, miRNA regulation, chromatin accessibility, DNA methylation, and histone modifications, revealing a coordinated multilayered reprogramming associated with metastatic progression. Meanwhile, by comparing metastatic rates between *Cs*-infected (*Cs*^+^) and non-infected (*Cs*^-^) HCC patients and combining these findings with *in vitro* functional assays, we demonstrated that *Cs* infection promotes the metastatic progression of HCC. These findings provide novel insights into parasite-driven metastasis and the adverse clinical outcomes observed in *Cs*-associated HCC, offering important implications for risk assessment and therapeutic strategies.

## 2 Methods

### 2.1 Samples collection

Liver tissue specimens were obtained from HCC patients undergoing radical resection at the Department of Hepatobiliary Surgery, Cancer Hospital Affiliated to Guangxi Medical University(Nanning, China). All patients had received no prior anticancer therapy and no history of other malignancies.

Tumor specimens exhibiting typical macroscopic features were excised from primary HCC nodules during surgery. Histopathological diagnosis was confirmed by hematoxylin and eosin (H&E) staining of tissue sections. Additionally, paired adjacent non-tumorous tissues were collected from areas at least 5 cm away from the tumor margin, confirmed to be free of tumor cells by histopathological examination. This study was approved by the Ethics Committee of the Cancer Hospital Affiliated to Guangxi Medical University (KY20251037) and complied with the ethical principles of the Declaration of Helsinki. Upon admission, Written informed consent was obtained from all patients prior to surgery.

### 2.2 Study population and data collection

A retrospective analysis was conducted on 1,239 treatment-naive HCC patients who underwent surgical resection between January 2013 and December 2024. Inclusion criteria were as follows: (i) Postoperative histopathological confirmation of HCC after surgical resection; (ii) No prior anticancer therapy; (iii) Absence of other malignancies; (iv)Availability of complete laboratory, histopathological, follow-up, and metastasis data. Diagnosis of *Cs* infection was based on one or more of the following: (i) preoperative stool examination revealing *Cs* eggs; (ii) preoperative imaging (MRI, CT, endoscopic ultrasound, or ultrasound) demonstrating *Cs* eggs or adult worms in intrahepatic bile ducts; (iii) intraoperative or postoperative pathology showing adult worms in the liver or gallbladder. For postoperative metastasis monitoring, patients underwent routine imaging studies (CT, MRI, or PET) and clinical evaluation to detect potential metastases, including those to extrahepatic sites (e.g., lungs, bones, lymph nodes) or other liver regions. Metastatic lesions are confirmed via tissue biopsy, including intraoperative or postoperative pathology examination of resected specimens to detect tumor cells in distant tissues.

### 2.3 Collection of the metastasis-related gene set

The metastasis-related genes were collected from genes reported in previous studies that are associated with HCC metastasis. Finally, 369 metastasis-related genes were obtained (**Supplementary Table S3**).

### 2.4 RT-qPCR

Total RNA was extracted from clinical tumor tissues of *Cs*^+^ HCC and *Cs*^−^ HCC patients using TRIzol reagent (Invitrogen, USA) according to the manufacturer’s protocol. Tissue specimens were powdered in liquid nitrogen before RNA isolation. Then, 1.0 μg of total RNA was reverse transcribed into complementary DNA (cDNA) using the Reverse Transcription Master Kit (Takara, Japan) according to the manufacturer’s instructions. Quantitative reverse-transcription PCR (RT-qPCR) was carried out on a qTOWER3 real-time PCR system (Jena, Germany) using TB Green Premix Ex Taq II FAST (2×) (Takara, Japan). The thermal profile comprised an initial denaturation at 95 °C for 30 s, followed by 39 cycles of 95 °C for 5 s and 60 °C for 30 s. Relative gene expression levels were normalized to GAPDH. ΔCt values were used for statistical analyses, and the 2^–ΔΔCt method was applied for data visualization. All reactions were performed in technical triplicate to ensure reproducibility. Differences between groups were assessed using a two-tailed Student’s t-test, with p < 0.05 considered statistically significant. The experiments were independently repeated three times to confirm consistency and reliability. Bulge-loop RT primers and qPCR primers specific for *SPP1*, *MMP2* and *VCAM1* were designed and synthesized by Sangon Biotech (Shanghai, China). Primer sequences are listed in **Supplementary Table S1**. Primer specificity was verified using the Basic Local Alignment Search Tool (BLAST) at the National Center for Biotechnology Information (NCBI) website (https://www.ncbi.nlm.nih.gov/).

### 2.5 Analysis of RNA−seq data

Raw reads were first processed with Trim Galore (v.0.6.10) (30) to remove adapter sequences and low-quality bases. The resulting high-quality reads were then aligned to the hg38 reference genome using Hisat2 (v.2.2.1) (31) under default settings. Read counts for each gene were generated using featureCounts (v.2.0.6) (32). Differential expression analysis was carried out with the DESeq2 package (v.1.44.0) (33) in R, considering genes with |Fold Change| > 2 and an adjusted p-value < 0.05 as significant. Functional enrichment of these differentially expressed genes was assessed through Gene Ontology (GO) and Kyoto Encyclopedia of Genes and Genomes (KEGG) analyses using the clusterProfiler R package (v.4.12.0) (34).

### 2.6 Analysis of miRNA−Seq

Raw sequencing reads were processed with Trim Galore (v0.6.10) (30) to filter out low-quality sequences and adapter contaminants, yielding high-quality clean data. These reads were then aligned to the hg38 reference genome using Hisat2 (v2.2.1) (35) under default settings. miRNA annotations in GFF3 format were acquired from miRBase (https://www.mirbase.org/). Expression levels of miRNAs were quantified using featureCounts (v2.0.6) (32) to generate an expression matrix. Differential expression analysis was performed using the R package DESeq2 (v1.44.0), with genes exhibiting |fold change| > 2 and a p-value < 0.05 considered statistically significant. Experimentally supported miRNA–target interactions were retrieved from miRTarBase (https://mirtarbase.cuhk.edu.cn/).

### 2.7 Analysis of ATAC−Seq data

Raw sequencing data were preprocessed with Trim Galore (v0.6.10) (30) to eliminate adapter sequences and low-quality reads, producing high-quality clean data. The resulting reads were aligned to the human reference genome (hg38) using Bowtie2 (v2.5.1) (36) under the parameters –very-sensitive -X 2000. PCR duplicates were marked and removed with Sambamba (v0.6.6) (37), and mitochondrial reads were excluded from downstream analysis. The aligned BAM files were converted into BigWig format using bamCoverage from DeepTools with RPKM normalization. Peak calling was performed using MACS2 with the settings -g hs –nomodel –shift -100 –extsize 200. Resulting peaks were visualized in the Integrative Genomics Viewer (IGV, v2.16.1) (38). Differential peak accessibility was assessed with the DESeq2 package (v1.44.0) (33) in R, applying thresholds of |Fold Change| > 2 and p < 0.05. *De novo* motif enrichment analysis was conducted using HOMER’s findMotifsGenome.pl script under default criteria (p < 0.01). Genomic annotation of peaks was performed with the annotatePeak function from the ChIPseeker package (v1.34.1) (39), with promoter regions defined as ±3 kb from the transcription start site (TSS). Gene activity scores were derived by counting reads in promoter-associated peaks per gene. Differential expression and activity were defined by |log2(Fold Change)| > 0.5 and p < 0.05.

### 2.8 Integrative analysis of ChIP−seq and ATAC−seq data

Given the limited availability of ChIP-seq data from primary hepatocellular carcinoma (HCC) samples—especially those associated with *C. sinensis* infection—we employed well annotated ChIP-seq datasets from HepG2 cells as the most suitable currently available proxy. We retrieved BigWig files from a HepG2 ChIP-seq study (GSE29611) available in the GEO database. Genomic coordinates were converted from hg19 to hg38 using CrossMap (v0.7.0) (40). Enrichment profiles from both ChIP-seq and ATAC-seq data were computed with the computeMatrix utility in DeepTools, and subsequently visualized via the plotHeatmap function. Representative genomic regions were displayed using the Integrative Genomics Viewer (IGV, v2.16.1) (33).

### 2.9 WGBS and oxWGBS data processing

Raw sequencing data were processed with Trim Galore (v0.6.10) (30) to remove adapter sequences and low-quality bases, yielding high-quality clean reads. These reads were aligned to the hg38 reference genome using BSMAP (v2.90) (41) with parameters “-p 10 -v 0.05”. PCR duplicates were removed using Sambamba (v0.6.6) (37). Methylation levels at individual CpG sites were quantified using the methratio.py script from the BSMAP package. Only CpG sites covered by at least 10 reads were retained for downstream analysis. Differentially methylated sites were called with the limma R package (v3.54.2), applying thresholds of |logFC| ≥ 0.5 and p < 0.05. Differentially methylated regions (DMRs) and differentially hydroxymethylated regions (DhMRs) were identified using Metilene (v0.2.8) under the following criteria: |Δβ| > 0.2, minimum of 10 CpG sites per region, and FDR < 0.05.

### 2.10 TCGA database analysis

Univariate Cox regression analysis was conducted using the R packages ‘survival’ (v3.8–3) and ‘survminer’ (v0.5.3) with data from the TCGA-LIHC cohort. Subsequent analyses were carried out using the ‘TCGAplot’ package (v7.0.1) within the same cohort. Specifically, gene_methylation_scatter was employed to assess the correlation between gene expression and promoter methylation; methy_kmplot was applied for survival analysis based on promoter methylation levels of individual genes; tcga_kmplot was used to evaluate the prognostic value of specific gene expression; gene_network_go facilitated the construction of a cnetplot visualizing connections between genes and associated GO terms; gene_gene_scatter enabled the visualization of gene-gene correlations through scatter plots within a specified cancer type; and gene_coexp_heatmap generated heatmaps along with GO enrichment analysis for genes positively or negatively co-expressed with a query gene in a particular cancer context.

### 2.11 Construction of the protein–protein interaction network

The protein–protein interaction (PPI) network was constructed to explore functional associations among proteins of different expressed metastasis-related genes between *Cs*^+^ and *Cs*^−^ HCC. The list of candidate proteins was submitted to the STRING database (version 11.5) to retrieve experimentally validated and predicted interactions. The resulting interaction data were imported into Cytoscape (v3.7.2) (42) for network visualization and further analysis.

### 2.12 LncRNA–miRNA–mRNA network build

Target genes of lncRNAs were predicted using miRCode (http://www.mircode.org/), while miRNA target genes were identified through miRTarBase (https://mirtarbase.cuhk.edu.cn/). Differentially expressed lncRNAs, miRNAs, and mRNAs were integrated to construct a lncRNA–miRNA–mRNA regulatory network, which was visualized using Cytoscape (v3.7.2) (42). A flowchart outlines the overall procedure, from the selection of differentially expressed transcripts to the assembly of the ceRNA network.

### 2.13 Data availability

The raw sequencing data generated in this study—including RNA-Seq, miRNA-Seq, WGBS-Seq, oxWGBS-Seq, and ATAC-Seq—have been deposited in appropriate public repositories. The RNA-Seq data are accessible through the NCBI Sequence Read Archive under BioProject accession number PRJNA1173109. The miRNA-Seq data has been deposited at SRA (PRJNA1168197). Both WGBS-Seq and oxWGBS-Seq datasets have been submitted to the NCBI Sequence Read Archive as well as the Gene Expression Omnibus (GEO) under accession number GSE284332. The ATAC-Seq data are available under GEO accession number GSE276855. Furthermore, publicly available ChIP-Seq data utilized in this study were retrieved from the GEO repository under accession number GSE29611.

### 2.14 Collection and preparation of *Cs* excretory/secretory products

*Cs* metacercariae were harvested from naturally infected Pseudorasbora parva captured in Hengxian County, Guangxi, China. Fish were processed by removing non-muscle tissues, deboning and mincing; tissues were then digested overnight at 37 °C in 0.8% pepsin with 0.2% HCl. The digestion slurry was filtered through a 60–80 mesh sieve, and live metacercariae were isolated microscopically and stored in phosphatebuffered saline (PBS) at 4 °C. Adult *C. sinensis* worms were recovered from the bile ducts of infected Sprague–Dawley(SD) rats, washed thoroughly with PBS containing 1% penicillin–streptomycin, and placed in phenol red–free 1640 medium (Solarbio, China) in glass dishes; the medium was renewed every 6–12 h. After 48 h of culture, the medium was pooled and clarified by centrifugation at 12,000 rpm for 30 min at 4 °C. The supernatant was dialyzed in PBS and concentrated either by sucrose-cushion ultracentrifugation or lyophilization depending on downstream application. Protein concentration and total yield were measured, aliquoted, and stored at −80 °C. Prior to use in downstream assays, *Cs*ESP preparations were sterilized by passage through a 0.22-µm pore-size filter.

### 2.15 Cell culture

Human HCC cell lines MHCC-97H were obtained from the Type Culture Collection of the Chinese Academy of Sciences (Shanghai, China). MHCC-97H cells were cultured in high-glucose Dulbecco’s Modified Eagle Medium (DMEM) (Gibco, USA) supplemented with 10% fetal bovine serum (FBS) (Wisent, Canada) and 1% PenicillinStreptomycin solution (Solarbio, China). In the experimental group, the concentration of *Cs*ESPs was set at 50 mg/mL, while the control group received an equivalent volume of 1×PBS (Gibco, USA) solution. Cells were maintained at 37°C in a humidified atmosphere containing 5% CO2 and 95% air.

### 2.16 Cell proliferation

Cell proliferation was measured using the Cell Counting Kit-8 (CCK8,UElandy, China) according to the manufacturer’s instructions. MHCC-97H cells were seeded in 96-well plates at a density of 2 × 10³ cells per well in 100 μL medium. Cells in the experimental group were treated with 50 mg/mL *Cs*ESPs, while control wells received an equal volume of 1× PBS. On Days 0, 1, 2, and3, the medium was replaced with 100 mL of serum-free medium in each well, followed by the addition of 10 μL of CCK8 reagent. Plates were incubated for 1 h at 37 °C, and absorbance at 450 nm was recorded using a microplate reader to determine relative cell proliferation.

### 2.17 Wound healing assay

MHCC-97H Cells were seeded in six-well plates at a density of 5 × 10^5^ cells per well and incubated for 24 h in DMEM supplemented with 10% heat-inactivated fetal bovine serum (FBS, Wisent, Canada) at 37 °C in a humidified incubator with 5% CO_2_. When the cells reached 80% to 90% confluence, the culture mediumwas removed, and the floating cells were washed twice with 1× PBS (Gibco, USA). Scratch wounds were created by drawing three horizontal lines per well using a 10 μL pipette tip, followed by washing with PBS to remove detached cells and debris. Cells were then cultured in medium containing 1% FBS and treated with either 50 mg/mL *Cs*ESPs (experimental group) or an equal volume of 1× PBS (control group). Cell migration into the wound area was imaged using a ZEISS Axio Vert.A1 light microscope immediately after treatment (0 h) and subsequently at 24 h intervals for up to 72 h. Scratch areas were analyzed at low magnification to quantify and compare migration rates between groups.

### 2.18 Transwell assay

The migration and abilities of MHCC-97H cells were evaluated using Transwell chambers (Costar, USA). Cells were pre-treated with 50 mg/mL *Cs*ESPs (experimental group) or an equal volume of 1× PBS (control group) and serum-starved for 24 h prior to the assay. For the migration assay, 1 × 10^6^ cells in 200 μL serum-free DMEM were seeded into the upper chamber. For the invasion assay, the upper chamber was pre-coated with Matrigel (Corning, USA) diluted 1:8 in serum-free DMEM. The lower chamber was filled with 600 μL DMEM containing 20% FBS as a chemoattractant. After 48 h incubation at 37 °C, cells that had migrated or invaded to the lower surface of the membrane were washed with PBS, fixed with 4% formaldehyde for 30 min, and stained with 0.5% crystal violet (Solarbio, China). Stained cells were imaged using a ZEISS Axio Vert.A1 microscope, and quantitative analysis was performed by counting cells in three randomly selected high-power fields per membrane.

### 2.19 Statistical analysis

All statistical analyses were performed using GraphPad Prism 9.5.0.Categorical variables, presented as counts or percentages, were compared using the Chi-square test, while continuous variables were analyzed using Student’s t-test or one-way ANOVA as appropriate. Experimental data represent means ± standard deviation (SD) from three independent experiments. Two-tailed p values < 0.05 were considered statistically significant.

## 3 Results

### 3.1 Clinical data suggests increased metastatic potential in *Cs*^+^ HCC

**Figure 1:**
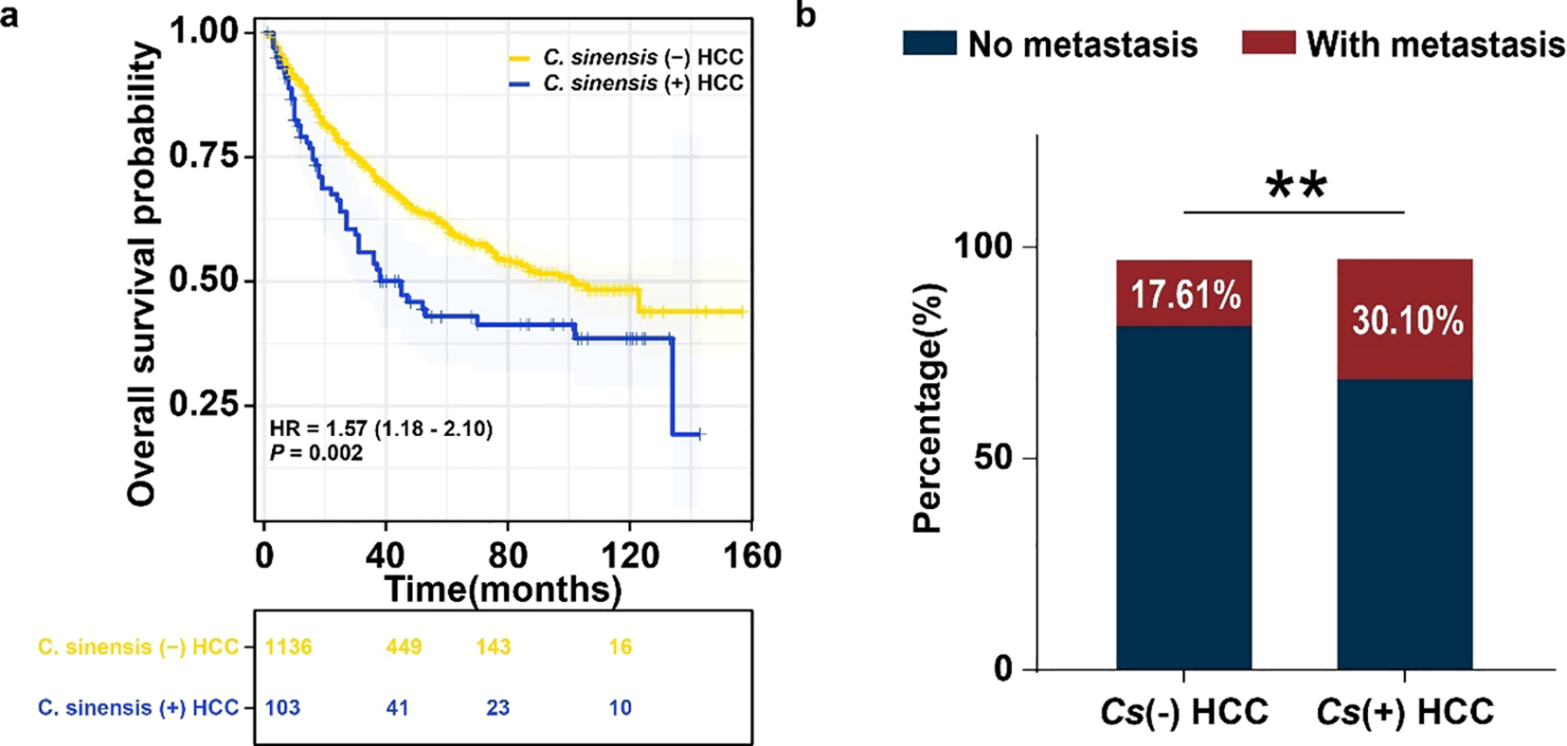
Retrospective clinical analysis of metastatic potential in *Cs*^+^ versus *Cs*^-^ HCC patients. **(a)** Kaplan–Meier curves showing the association between *C. sinensis* infection and overall survival. **(b)** Metastasis rate comparison between *Cs*^+^ (n = 103) and *Cs*^-^ (n = 1,136) HCC cases. ** representing *P* < 0.01.

We began by examining prognostic differences between *C. sinensis*-positive (*Cs*^+^) and *C. sinensis*-negative (*Cs*^-^) HCC patients. A survival analysis was performed on 1,239 treatment-naïve HCC patients who underwent surgical resection between January 2013 and December 2024. The results indicated that the median OS for the *Cs*^+^ HCC group was 45 months compared to 102 months for the *Cs*^-^ HCC group (hazard ratio (HR) = 1.57, 95% confidence interval (CI):1.18 to 2.10;*P* = 0.002)(**Figure 1a**). *Cs*^+^ status was associated with significantly poorer overall survival. The information on all patients was listed in **Supplementary Table S2**. We then performed a separate retrospective analysis within the same cohort, focusing on metastasis and *C. sinensis* infection. This analysis revealed a significantly higher metastasis rate among *Cs*^+^ patients compared to *Cs*^-^ patients (30.10% vs. 17.61%, p < 0.01), underscoring a clear association between *C. sinensis* infection and metastatic progression in HCC (**Figure 1b**).

### 3.2 RNA-seq analysis of metastasis-related genes between *Cs*^+^ and *Cs*^−^ HCC

**Figure 2:**
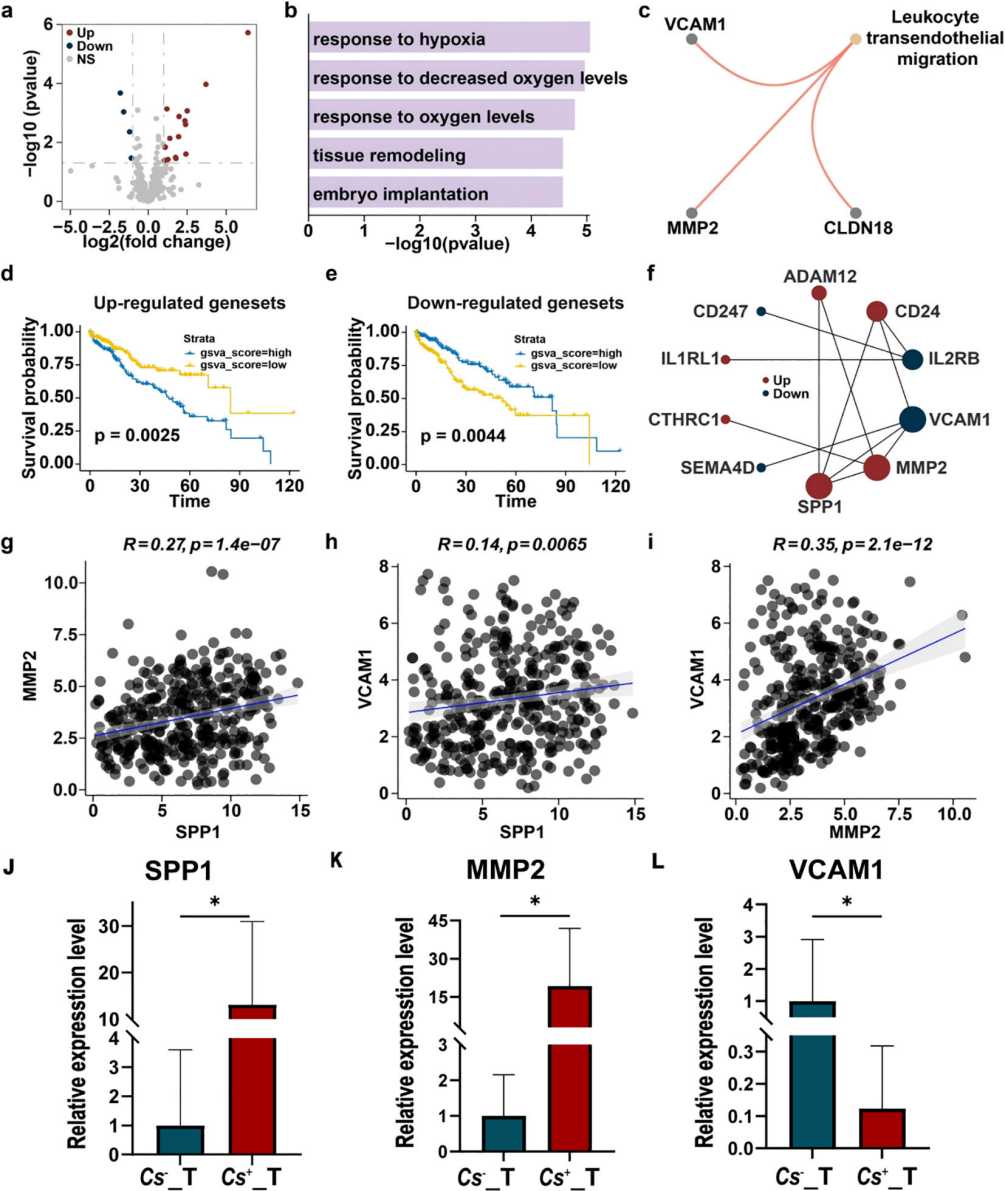
*C. sinensis* infection alters the expression profile of metastasis-associated genes in HCC tumors. **(a)** Volcano plot indicating down- and up-regulated metastasis-related genes in *Cs*^+^ versus *Cs*^-^ HCC tumors. **(b, c)** GO term **(b)** and KEGG pathway **(c)** enrichment analyses of differentially expressed metastasis-related genes between *Cs*^+^ and *Cs*^-^ HCC tumors. **(d, e)** Kaplan–Meier survival analysis evaluating the prognostic value of up-regulated **(d)** and down-regulated **(e)** metastasis-related gene sets from *Cs*^+^ HCC tumors within the TCGA-LIHC cohort. **(f)** Protein–protein interaction (PPI) network of metastasis-related genes differentially expressed in *Cs*^+^ HCC tumors. **(g-i)** Spearman correlation analysis examining relationships among three hub genes. **(j-l)** RT-qPCR validation of differentially expressed metastasis-related genes in *Cs*^+^ and *Cs*^−^ HCC tumor samples. Relative expression levels calculated by the 2^–ΔΔCt method, while statistical significance was assessed using ΔCt values. Data were presented as means ± SD; n = 5. Student’s t-test was used.

Given the association between *C. sinensis* infection and enhanced metastatic potential in HCC, we sought to further investigate the underlying molecular mechanisms. To assess alterations in metastasis-related pathways, we compiled a set of 369 metastasis-associated genes and evaluated their expression in 10 C*. sinensis*-positive (*Cs*^+^) and 10 C*. sinensis*-negative (*Cs*^-^) HCC tumor samples from our previous cohort. Differential expression analysis identified 20 metastasis-related genes with significant changes, including 4 downregulated and 16 upregulated genes (**Supplementary Table S4**). These results are summarized in a volcano plot (**Figure 2a**). Gene Ontology (GO) enrichment analysis indicated that these genes are implicated in biological processes including response to hypoxia and variations in oxygen levels (**Figure 2b**). KEGG pathway analysis further revealed enrichment in leukocyte transendothelial migration, hinting at an altered immune microenvironment triggered by *C. sinensis* infection (**Figure 2c**). Subsequent prognostic evaluation using the TCGA-LIHC cohort demonstrated that elevated expression of the upregulated gene set and reduced expression of the downregulated gene set were both significantly correlated with poorer survival outcomes (**Figures 2d, e**). Additionally, prognostic analysis of individual differentially expressed metastasis-related genes identified *ADAM1*, *CTHRC1*, *PMAIP1*, *SEMA4D*, *SPP1*, and *STC2* as significantly associated with patient prognosis (**Supplementary Figure S1**). To explore functional interactions among these genes, a protein–protein interaction (PPI) network was constructed (**Figure 2f**), highlighting *SPP1*, *MMP2*, and *VCAM1* as top hub genes within the differentially expressed metastasis-related gene set. Correlation analysis showed a significant positive relationship among these three genes (**Figures 2g–i**). Finally, we identified genes that were positively and negatively co-expressed with these hub genes in the TCGA-LIHC cohort (**Supplementary Figure S2**).We experimentally validated the transcriptomic findings by performing RT–qPCR on *Cs*^+^ and *Cs*^-^ HCC tumor samples (n = 5 per group). Consistent with the RNA-seq results, the expression of *SPP1*, *MMP2*, and *VCAM1* was significantly higher in *Cs^+^* tumors compared with *Cs*^-^ tumors (*SPP1*: p = 0.0102; *MMP2*: p = 0.0276; *VCAM1*: p = 0.0259; **Figure 2j–l**). These findings confirm the upregulation of key metastasis-related hub genes in *Cs^+^* HCC tumors and further support a role for *Cs* infection in promoting a pro-metastatic transcriptional program.

### 3.3 Identification of differentially expressed miRNAs interacting with metastasis-related genes in *Cs*^+^ HCC

**Figure 3:**
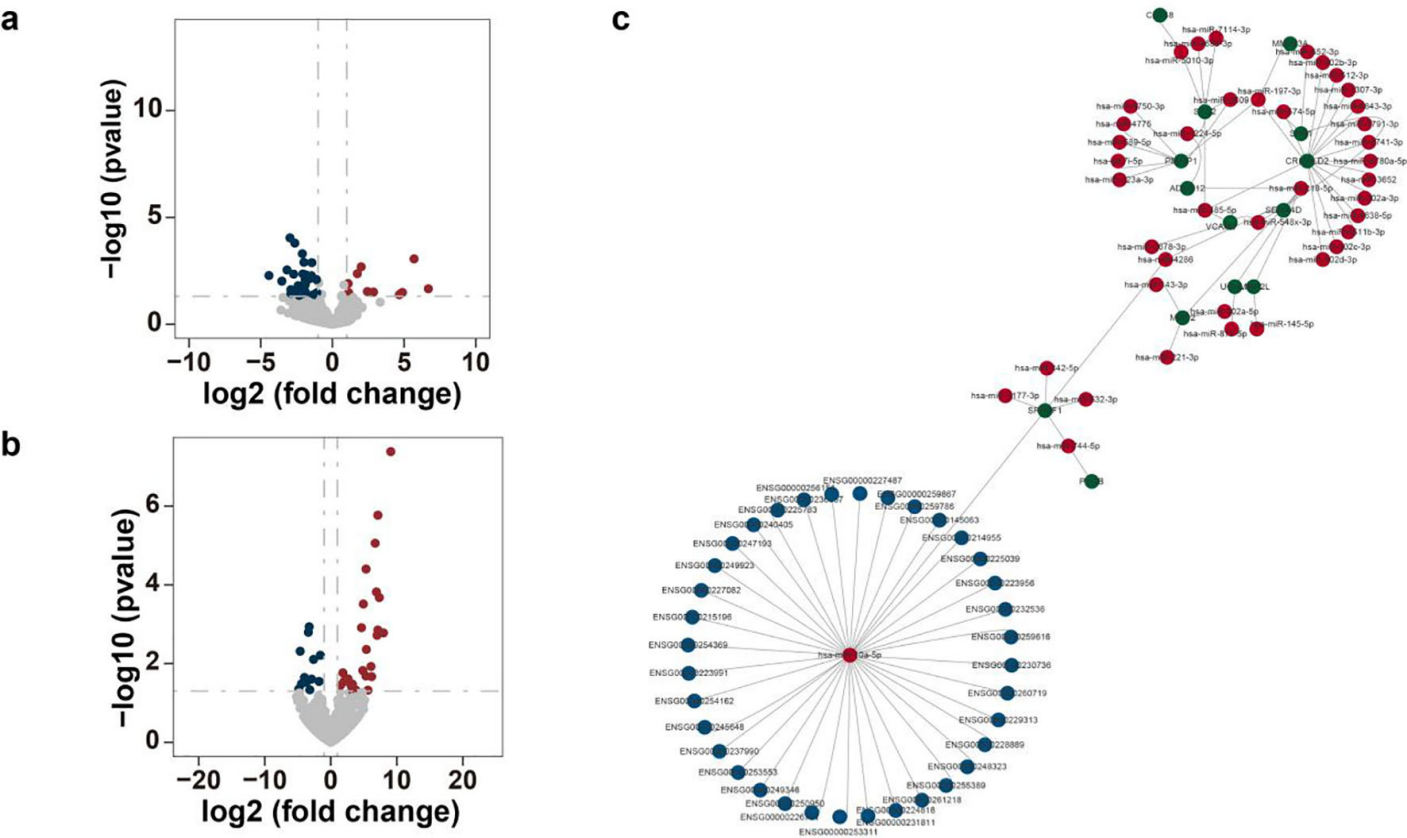
Analysis of miRNA expression profiles in *Cs*^+^ HCC. **(a)** Volcano plot of differentially expressed miRNAs interacting with metastasis-related genes in *Cs*^+^ versus *Cs*^-^ HCC tumors. **(b)** Volcano plot of differentially expressed lncRNAs interacting with metastasis-related genes in *Cs*^+^ versus *Cs*^-^ HCC tumors. **(c)** Computationally constructed ceRNA network illustrating potential interactions among lncRNAs (blue nodes), miRNAs (red nodes), and metastasis-related mRNAs (green nodes) in the context of *C. sinensis* infection.

To elucidate the regulatory mechanisms driving distinct responses to *C. sinensis* infection, we compared miRNA expression profiles between *Cs*^+^ and *Cs*^-^ HCC tumors. We identified 12 up-regulated and 29 down-regulated miRNAs interacting with metastasis-related genes in *Cs*^+^ tumors relative to *Cs*^-^ controls (**Figure 3a**; **Supplementary Table S5**). Given that lncRNAs can act as miRNA sponges—modulating mRNA expression and influencing cellular physiology—we also analyzed differentially expressed lncRNAs that interact with metastasis-related genes. This analysis revealed 25 up-regulated and 12 down-regulated lncRNAs in *Cs*^+^ HCC (**Figure 3b**; **Supplementary Table S6**).

To further decode the complex cross-talk among lncRNAs, miRNAs, and mRNAs, we constructed a competing endogenous RNA (ceRNA) network. This network revealed that 36 differentially expressed lncRNAs potentially regulate 41 miRNAs, which in turn target 14 mRNAs, uncovering a multi-layer regulatory architecture. The ceRNA network comprises 91 nodes and 93 edges, reflecting the intricate interactions among these RNA species (**Figure 3c**).

### 3.4 ATAC-seq profiling of chromatin accessibility in metastasis-related genes between *Cs*^+^ and *Cs*^-^ HCC

**Figure 4:**
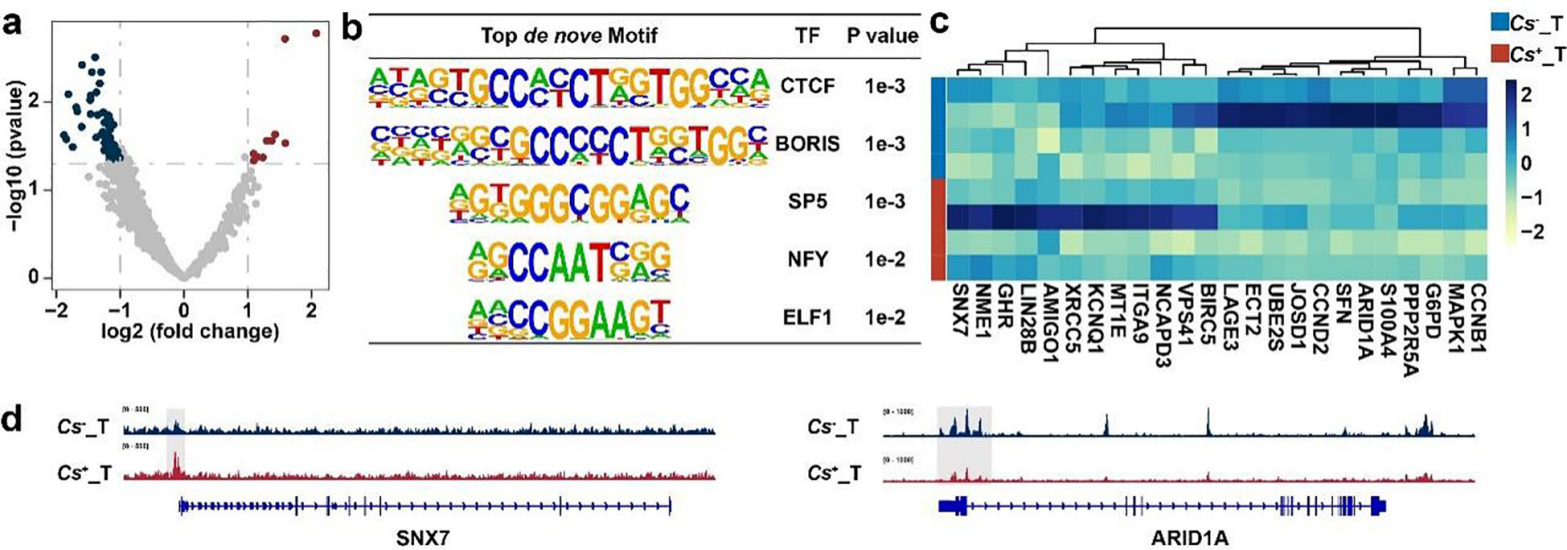
Alterations in chromatin accessibility landscape of metastasis-related genes following *C. sinensis* infection in HCC. **(a)** Differential chromatin accessibility in promoter regions of metastasis-related genes between *Cs*^+^ and *Cs*^-^ HCC tumors. **(b)** Top *de novo* motifs enriched within differential chromatin accessibility regions in the promoters of metastasis-related genes. **(c)** Heatmap of gene activity scores for regions with differential chromatin accessibility in metastasis-related genes. **(d)** IGV browser tracks displaying representative differential peaks near metastasis-related genes.

Recognizing the significant contribution of metastasis to epigenetic modulation, we next examined chromatin accessibility patterns at promoters of metastasis-related genes using ATAC-seq data from our previous study, which included 4 *Cs*^+^ and 4 *Cs*^-^ HCC tumors. Comparative analysis revealed 10 regions with increased accessibility and 61 regions with decreased accessibility in *Cs*^+^ tumors relative to *Cs*^-^ samples (**Figure 4a**; **Supplementary Table S7**). *De novo* motif enrichment analysis of these differentially accessible regions identified the top five significantly enriched motifs: CTCF, BORIS, SP5, NFY, and ELF1 (**Figure 4b**). Gene activation scores were subsequently computed for genes linked to these promoter accessibility changes (**Supplementary Table S8**). From these scores, 24 differentially expressed genes were discerned and visualized in a heatmap (**Figure 4c**). Lastly, representative chromatin accessibility profiles near metastasis-related genes were illustrated (**Figure 4d**).

### 3.5 ChIP-seq analysis of histone modifications in metastasis-related genes between *Cs*^+^ and *Cs*^−^ HCC

**Figure 5:**
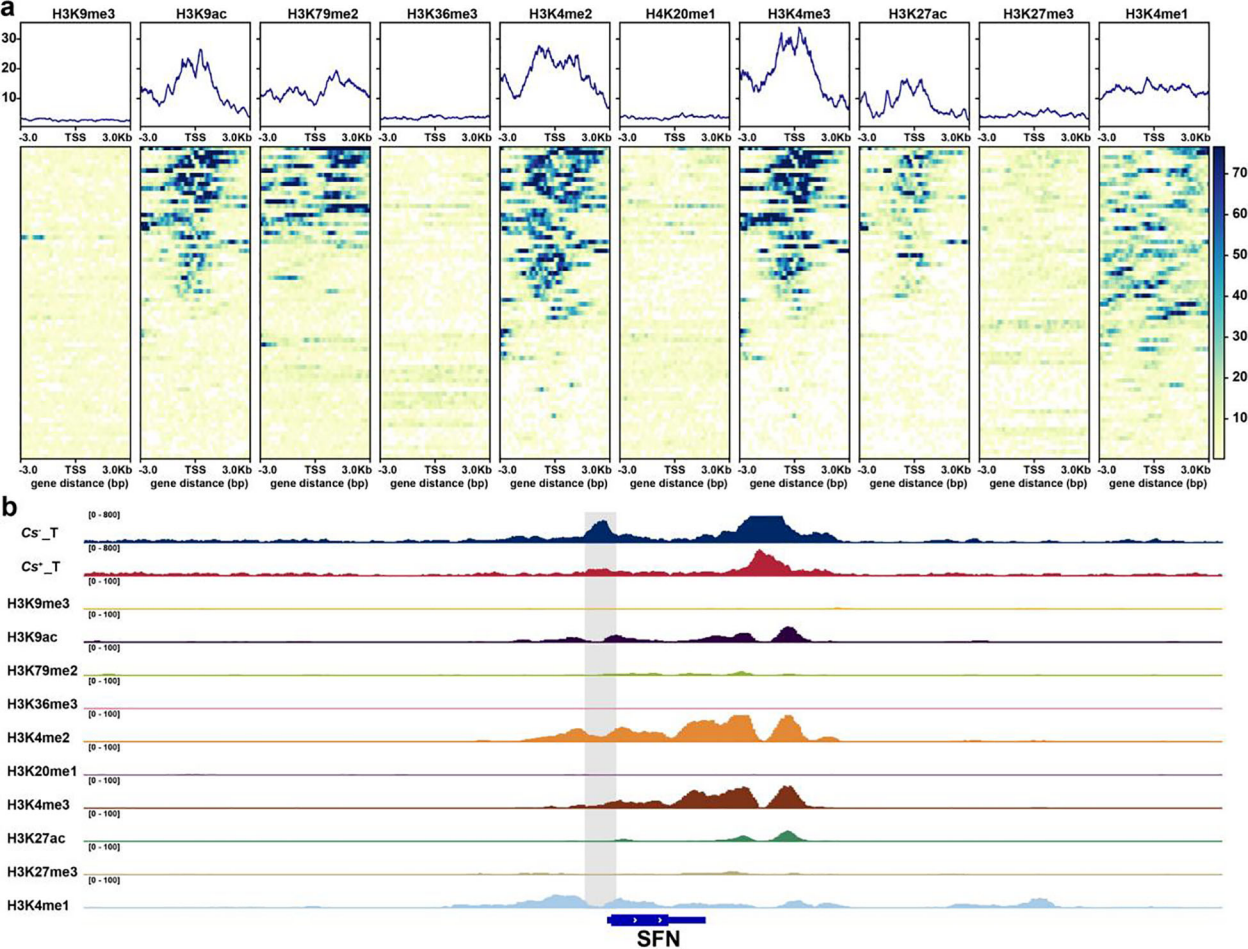
Integrative analysis of ATAC-Seq and public ChIP-Seq data from *Cs*^+^ and *Cs*^-^ HCC tumors. **(a)** Heatmap of histone modification signals overlapping differential chromatin accessibility regions in metastasis-related genes. **(b)** IGV visualization of representative ATAC-Seq and ChIP-Seq profiles in *Cs*^+^ versus *Cs*^-^ HCC tumors.

Histone modifications are known to play a crucial role in modulating chromatin accessibility. To investigate their impact in the context of metastasis induced by *C. sinensis* infection, we analyzed publicly available ChIP-seq data from the HepG2 cell line, encompassing major histone marks including H3K9me3, H3K9ac, H3K79me2, H3K36me3, H3K4me2, H4K20me1, H3K4me3, H3K27ac, H3K27me3, and H3K4me1. A heatmap was generated to visualize the enrichment patterns of these histone modifications within metastasis-associated differential chromatin accessibility regions between *Cs*^+^ and *Cs*^-^ HCC tumors. Notably, H3K9ac, H3K79me2, H3K4me2, H3K4me3, H3K27ac, and H3K4me1 exhibited strong associations with differential accessibility regions linked to metastasis-related genes (**Figure 5a**). We further displayed integrated ATAC-seq and ChIP-seq profiles across representative genomic regions to illustrate these relationships (**Figure 5b**).

### 3.6 Altered methylation regulates metastasis-related gene expression

**Figure 6:**
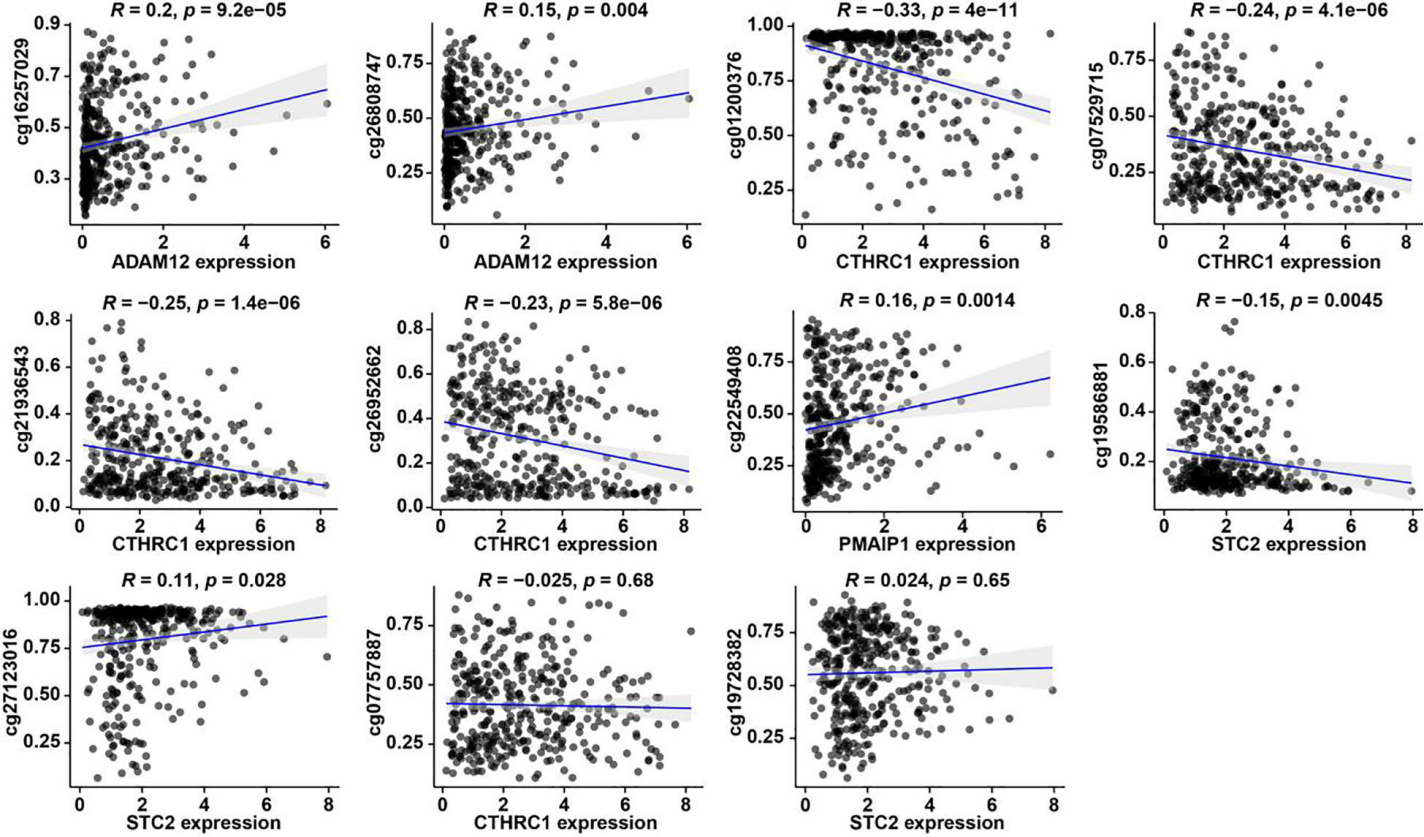
Relationship between DNA methylation and expression levels of metastasis-related genes in the TCGA-LIHC cohort.

To further investigate metastasis-associated epigenetic alterations induced by *C. sinensis* infection, we profiled DNA methylation and hydroxymethylation patterns using whole-genome bisulfite sequencing (WGBS) and oxidative bisulfite sequencing (oxWGBS) in four *Cs*^+^ and four *Cs*^-^ HCC tumors from our earlier work. We compared differentially methylated CpG sites within promoter regions between the two groups and extended this analysis to identify differential methylated regions (DMRs) and differential hydroxymethylated regions (DhMRs) linked to metastasis-related genes. However, no significant DMRs or DhMRs were detected near differentially expressed metastasis-related genes associated with prognosis in *Cs*^+^ HCC. We subsequently evaluated the relationship between promoter DNA methylation and the expression levels of these prognosis-relevant metastasis-related genes within the TCGA-LIHC cohort (**Figure 6**). This analysis identified 9 CpG sites exhibiting a strong correlation with gene expression, implying that *C. sinensis* infection may promote metastatic changes via DNA methylation mechanisms, potentially leading to poorer HCC outcomes (**Figure 5a**).

### 3.7 Experimental evidence that *C. sinensis* infection promotes HCC metastasis

**Figure 7:**
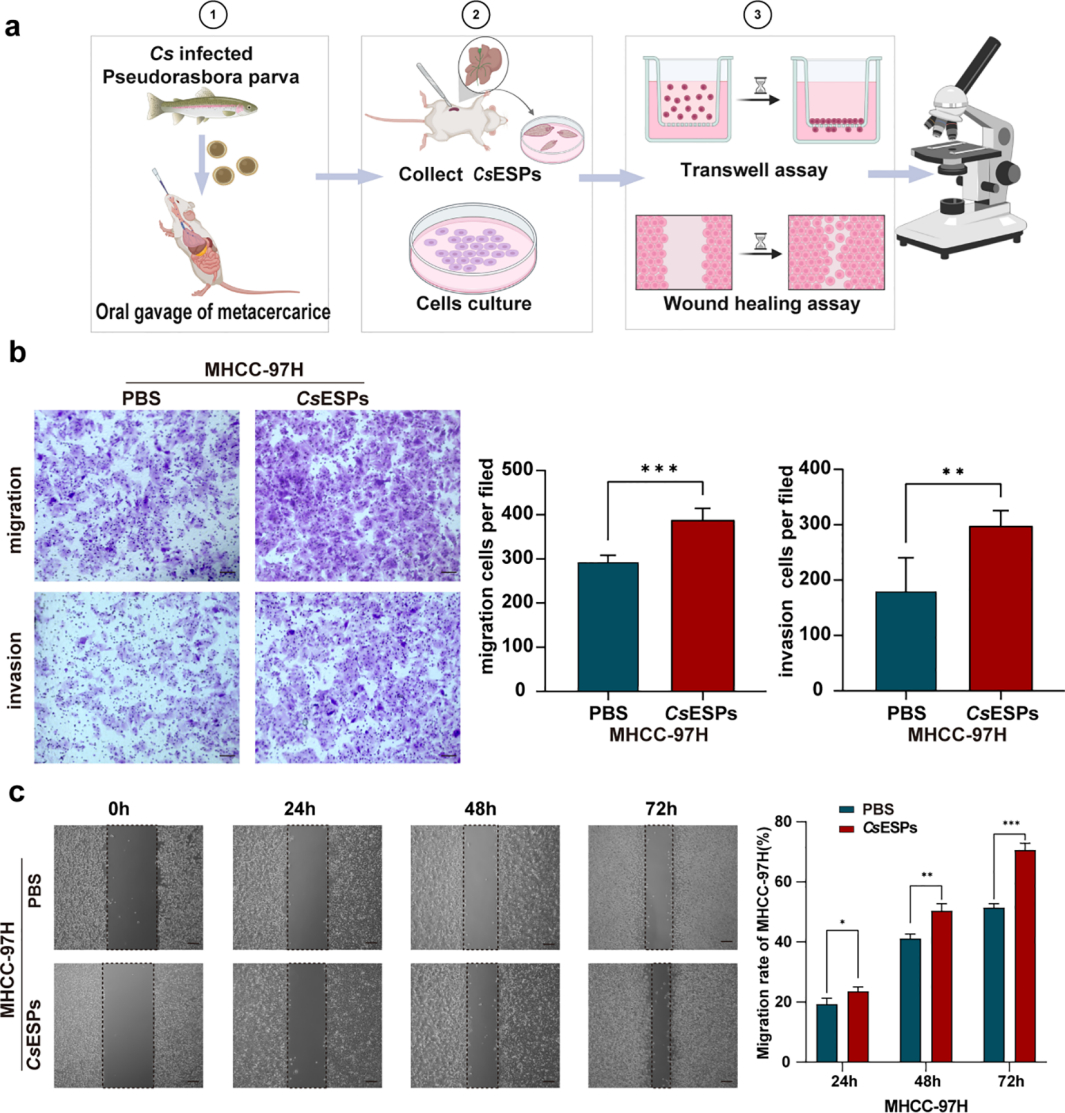
Experimental evidence that *C. sinensis* infection promotes HCC metastasis. **(a)** Schematic overview of the experimental design in this study. Created with BioRender.com. **(b)** Transwell assay of MHCC-97H cells co-cultured with *Cs*ESPs or PBS (Scale bar: 200 mm) (n = 3). **(c)** The wound-healing of MHCC-97H cells co-cultured with *Cs*ESPs or PBS (Scale bar: 50 mm) (n = 3). **(b, c)** Data are represented as mean± SD, *p < 0.05, **p < 0.01, ****p < 0.0001.

Given that both clinical data and multi-omics analyses indicated that *Cs* infection promotes metastasis-related alterations in HCC, we further validated *in vitro* whether *Cs*ESPs directly enhance the migratory and invasive capacities of HCC cells. As outlined in the experimental workflow (**Figure 7a**), MHCC-97H cells were co-cultured with either *Cs*ESPs or PBS as a control, and their migration and invasion were subsequently assessed. Transwell assays revealed that *Cs*ESPs significantly increased both the migration and invasion of MHCC-97H cells compared with the PBS control group (**Figure 7b**). Quantitative analysis showed markedly higher numbers of migrating (p < 0.001) and invading (p < 0.01) cells in the *Cs*ESPs-treated group than in controls. Consistent with these findings, wound-healing assays demonstrated that *Cs*ESPs significantly accelerated gap closure in MHCC-97H cells at 24, 48, and 72 hours (**Figure 7c**). Quantification of wound closure confirmed that migration rates in the *Cs*ESPs group exceeded those of the PBS control group at all time points (24 and 48 hours: p < 0.05; 72 hours: p < 0.001). Data are presented as mean ± SD of three independent experiments; statistical significance was determined by two-tailed Student’s t-test. Collectively, these results demonstrate that *Cs*ESPs markedly promote the migratory and invasive behavior of HCC cells *in vitro*, supporting a potential role for *Cs* infection in enhancing the metastatic potential of HCC.

## 4 Discussion

Currently, HCC remains one of the most prevalent malignant tumors in China, accounting for approximately 47% of new cases worldwide (43). *Cs* is widely endemic in southern China (44, 45). In *Cs*-endemic regions such as Guangxi, approximately 16% of HCC patients exhibit concurrent infection, with infection intensity positively correlated with HCC incidence (39). Previous studies have demonstrated that *Cs* infection is closely associated with poor prognosis in HCC patient (25, 46, 47). Consistently, our recent retrospective clinical study revealed that *Cs*-infected HCC patients exhibit significantly shorter median OS, accompanied by markedly higher metastasis rates. However, the precise mechanisms underlying this phenomenon remain unclear. Our analyses revealed 20 differentially expressed metastasis-associated genes (including the hub genes *SPP1*, *MMP2*, and *VCAM1*), 41 interacting miRNAs, and 71 promoter-accessible chromatin regions, with expression changes significantly correlated with high metastatic potential and poor prognosis in *Cs^+^* HCC patients. *In vitro* experiments confirmed that *Cs*-related stimulation enhances migration, invasion, and angiogenic potential of HCC cells, suggesting that *Cs* infection acts as a key driver of tumor metastasis. These findings provide a foundation for understanding *Cs*-associated HCC metastasis and for developing strategies to improve survival outcomes, overcome drug resistance, and guide individualized therapy.

Among the metastasis-associated genes identified, *SPP1*, *MMP2*, and *VCAM1* occupy central positions and show significant correlations in *Cs*^+^ HCC. RT-qPCR validated the upregulation of *SPP1* and *MMP2*, whereas *VCAM1* displayed a downregulation trend. Previous studies have reported that *SPP1* and *MMP2* are upregulated in multiple highly metastatic cancers, including HCC, lung cancer, and gastric cancer (48). And recent studies in breast cancer have shown that *SPP1* is essential for maintaining mesenchymal cell fate (49). Upon binding to CD61, SPP1 activates the NF-κB–BMP2–GREM1 axis, ultimately forming a paracrine positive feedback loop across cellular subpopulations, the core function of which is to sustain mesenchymal cell fate with high migratory and invasive potential, thereby promoting metastasis (49). *SPP1* also promotes tumor immune evasion by activating the PI3K–AKT–mTOR pathway and recruiting MDSCs and Tregs to establish an immunosuppressive microenvironment, thereby facilitating tumor metastasis (50, 51). Moreover, *SPP1* stimulates neutrophils to generate neutrophil extracellular traps (NETs), thereby establishing a pre-metastatic niche (PMN) in lung tissue that “captures” circulating tumor cells and facilitates lung metastasis (52, 53), consistent with our KEGG pathway analysis of “leukocyte transendothelial migration”. *MMP2* degrades the extracellular matrix (ECM) and basement membrane, promotes EMT and angiogenesis, and establishes a positive feedback loop with cancer-associated fibroblasts (CAFs) to remodel an immunosuppressive microenvironment (54, 55). In cholangiocarcinoma, it has been demonstrated that *Cs* infection activates *MMP2* via induction of EMT, ultimately driving local invasion and distant metastasis (56). Given the observed upregulation of *MMP2* in our HCC samples, we speculate that *MMP2* may similarly facilitate ECM-mediated metastasis in *Cs*^+^ HCC. *VCAM1* mediates circulating tumor cell (CTC) adhesion, tumor–stromal interactions, and metastasis formation across various cancers, promoting endothelial cell proliferation and angiogenesis (57, 58). *VCAM1* mRNA and protein levels are markedly elevated in multiple cancers relative to normal tissues, although its role in HCC remains poorly understood (59). In contrast, we observed downregulation of *VCAM1* in *Cs*^+^ HCC tumors, a phenomenon previously reported during iCCA metastasis (60). We hypothesize that *Cs*^+^ HCC may exhibit high *VCAM1* expression in early-stage *in situ* tumors to facilitate local adhesion, whereas *VCAM1* is downregulated during distant dissemination to decrease matrix adhesion (61), enabling tumor cells to detach more easily from the primary site and promote metastasis. Beyond transcriptional regulation, our multi-omics analysis highlights the pivotal role of epigenetic modifications in controlling chromatin accessibility. To further explore the regulatory mechanisms of metastasis-associated genes, we compared ChIP-seq data from *Cs*^+^ and *Cs*^^-^^ HCC samples. Results revealed significant enrichment of active histone acetylation marks, including H3K9ac and H3K27ac, in enhancer and promoter regions, closely associated with elevated expression of canonical metastasis-driving genes such as *ZEB1*, *MMP2*, and *IGF-1 (*62, 63). These modifications function not only as markers of chromatin accessibility but also as central epigenetic signals driving the acquisition of invasive and metastatic phenotypes in tumor cells. As observed in other cancer models, they likely amplify *Cs* infection-induced HCC progression by reshaping the epigenetic landscape of enhancers and promoters (64, 65). This enhances transcription factor binding efficiency and maintains elevated expression of metastasis-associated genes, thereby facilitating tumor cell migration, invasion, and distant metastasis.

Compared with previous studies on *Cs* infection-driven progression in HCC or cholangiocarcinoma (20, 66), we similarly observed poorer clinical outcomes in *Cs*-infected patients. However, unlike these studies, our analysis leveraged recent clinical data and specifically focused on metastasis in HCC, a hallmark of malignant tumors and a key determinant of prognosis (6). Our study confirmed that in *Cs^+^* HCC, *SPP1*, *MMP2*, and *VCAM1* are differentially expressed. Importantly, we further observed that these three genes occupy hub positions in the protein–protein interaction (PPI) network, suggesting potential mutual regulation, a finding not reported in previous studies. Notably, *VCAM1* remains largely unexplored in HCC. Previous studies have shown that *Cs*-induced chronic inflammation and fibrosis can activate the VEGF/angiopoietin signaling pathway to promote neovascularization (24), while our novel finding suggests that *Cs* may regulate *VCAM1* expression to facilitate endothelial cell proliferation and angiogenesis-mediated metastasis. Importantly, our study provides a novel multi-omics perspective on *Cs*-driven HCC, integrating transcriptomic, epigenetic, and chromatin accessibility data. These findings offer mechanistic insights into *Cs*-driven HCC metastasis and highlight potential targets for precision intervention.

Nonetheless, our study has several limitations. As a single-center retrospective analysis, selection bias cannot be excluded. Most patients had chronic viral hepatitis, including HBV or HCV, and many also presented with additional hepatic comorbidities such as cirrhosis of various etiologies, alcohol-related liver disease, and metabolic-associated fatty liver disease (MAFLD/NAFLD). These conditions may reshape the hepatic microenvironment through fibrosis, inflammation, and extracellular matrix remodeling, and thus constitute potential factors that promote metastatic progression in HCC (67–72). Future prospective multi-center studies with standardized inclusion criteria and more robust confounder-adjustment strategies, such as multivariable regression or propensity score techniques, are warranted to further validate our findings and improve their generalizability. In addition, *Cs* infection is typically chronic and, under prolonged exposure, can induce sustained inflammation and bile duct injury, which may also influence HCC metastatic progression (15, 73, 74). However, the present study assessed only infection status and did not capture information on infection duration, severity, or reinfection, all of which warrant investigation in future prospective cohort studies. Furthermore, the sample size of our study is relatively modest, which may limit the generalizability of our findings. For instance, the multi-omics datasets—particularly the ATAC-seq analysis—were based on a relatively small number of paired samples, which may reduce statistical power. Therefore, our analyses were designed to identify potential regulatory trends, with an emphasis on the integrative interpretation of chromatin accessibility, transcriptomic alterations, and clinical relevance. In addition, the limited sample size in the female *Cs*-associated HCC subgroup may introduce potential selection bias. Expanding the sample size in future studies will be essential to strengthen the validity and overall reliability of our findings. Notably, no significant DMRs/DhMRs were identified in our analysis. This may partly be attributed to the high sensitivity of DMR detection to sample size and statistical power, as the limited cohort size together with stringent multiple-testing correction may increase the risk of false-negative findings (75). Beyond technical considerations, epigenetic regulation is inherently hierarchical, and metastatic phenotypes may arise from regulatory layers beyond DNA methylation, such as chromatin accessibility, transcription factor activity, and histone modifications, which are not necessarily reflected by region-level methylation changes (76, 77). Another limitation of this study is that, although significant correlations were observed among metastasis-associated genes, histone modifications, and DNA methylation, the evidence presented is primarily associative rather than mechanistic. The predicted ceRNA network was constructed based on integrated computational prediction and correlation analyses and suggests potential lncRNA–miRNA–mRNA regulatory axes involved in *Cs*-driven metastatic progression (78, 79). However, these interactions do not represent definitive regulatory mechanisms and require further functional validation, such as luciferase reporter assays and gain- or loss-of-function experiments, to establish causality (78). In addition, the *in vitro* functional analyses in this study were conducted using the highly metastatic MHCC-97H cell line (80, 81). Consequently, the pro-invasive and pro-metastatic effects induced by *Cs*ESPs observed here may not fully capture the molecular heterogeneity across different HCC subtypes. Validation in additional HCC cell lines with distinct molecular backgrounds, such as HCCLM3, HepG2, or Huh7, is warranted to assess the generalizability of our findings. Moreover, future investigations should employ *in vivo* models and advanced genetic manipulation approaches, including gene knockout or knockdown strategies and targeted genome-editing techniques such as CRISPR/Cas9 and RNA interference (RNAi), to further validate the causal roles of key genes and epigenetic modifications and to elucidate the molecular mechanisms through which *Cs* infection promotes HCC metastasis (82).

Furthermore, single-cell transcriptomics (scRNA-seq) and spatial transcriptomics have emerged essential technologies for deciphering tumor heterogeneity, cell–cell interactions, and spatial organization within the microenvironment (83, 84). The combined application of scRNA-seq with scATAC-seq, spatial transcriptomics, and high-resolution multiplex immunohistochemistry (mIHC) enables the identification of the origin, activation status, and spatial colocalization of *Cs* infection-associated metastatic genes with immune or stromal cells across cellular subpopulations and spatial dimensions (85). This strategy facilitates a more precise reconstruction of the parasite-induced pro-metastatic environment and its epigenetic regulatory networks. Collectively, these approaches hold promise for mechanistically elucidating the comprehensive landscape of parasite-mediated tumor metastasis, providing novel insights for early intervention and targeted therapies in HCC.

## 5 Conclusions

In summary, our findings demonstrate that *Cs* infection promotes HCC metastasis by upregulating metastasis-associated genes and remodeling the epigenetic landscape. Multi-omics analyses provide mechanistic insights into parasite-driven tumor progression and highlight potential targets for early intervention to improve patient outcomes. These results should be validated in future larger multicenter studies, and the underlying mechanisms of metastasis warrant further investigation.

## Data Availability

The datasets presented in this study can be found in online repositories. The names of the repository/repositories and accession number(s) can be found in the article/**Supplementary Material**.
