# Supplementary material for: The metastasis landscape of Clonorchis sinensis-associated hepatocellular carcinoma: an integrated multi-omics and clinical study
Source: Front Immunol. 2026 Jan 28;17:1723156. doi: 10.3389/fimmu.2026.1723156 (PMC12891100; doi:10.3389/fimmu.2026.1723156)
Supplement: Supplementary file 1 [file Table1.docx]

**Supplymentary figures**


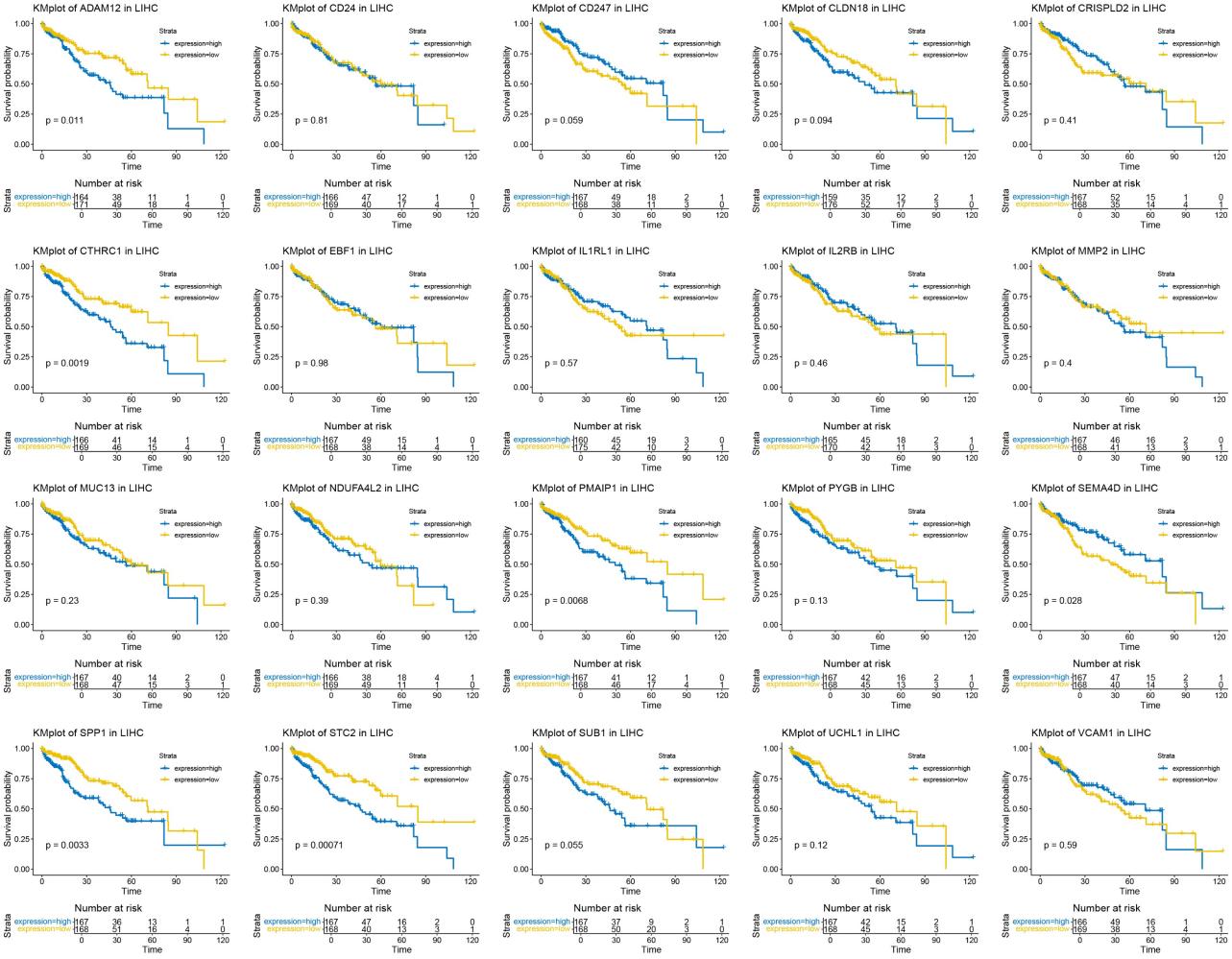


**Fig. S1.** Kaplan–Meier survival curves depicting the association between expression levels of metastasis-related genes differentially expressed in *Cs*⁺ HCC tumors and clinical outcomes in the TCGA-LIHC cohort.


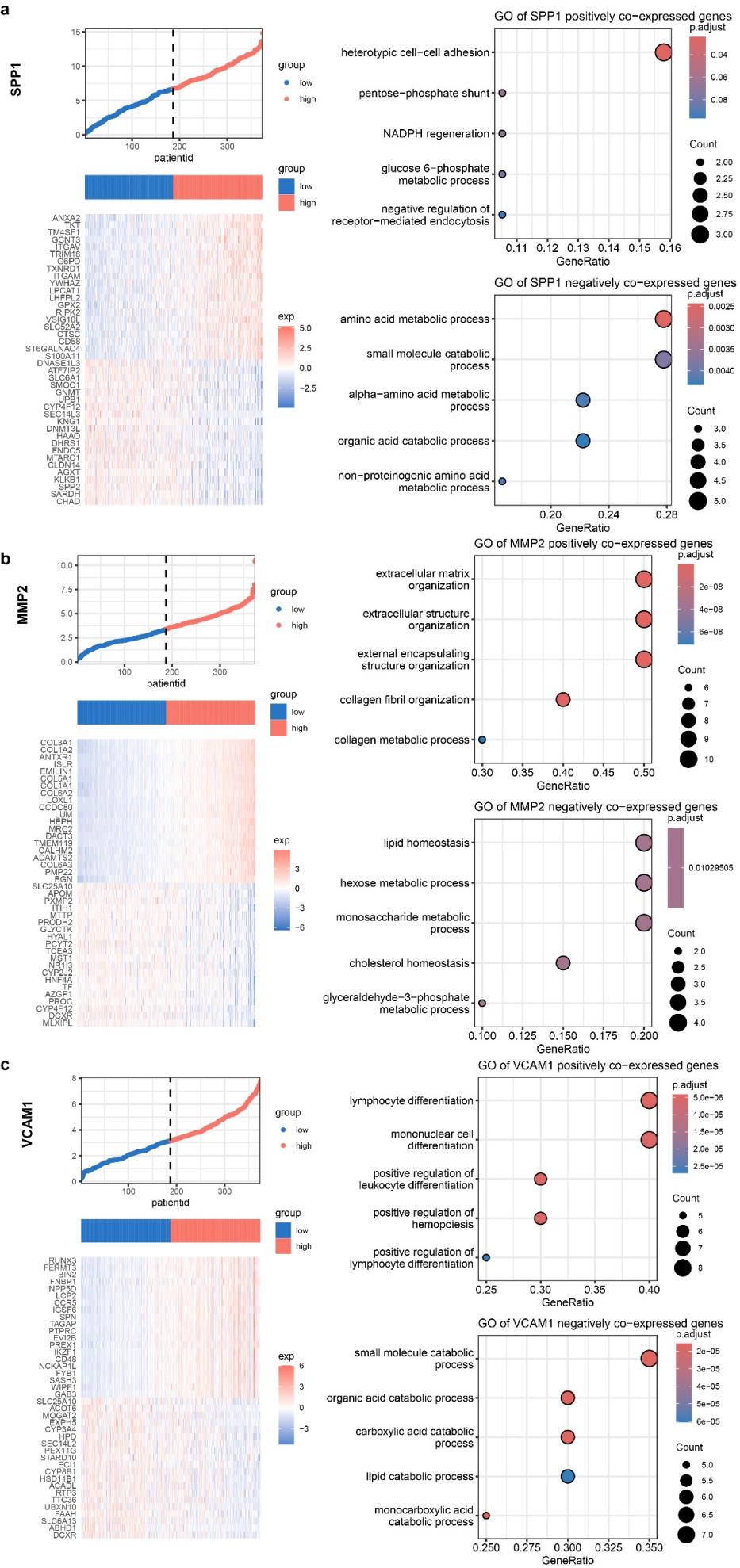


**Fig. S2.** Heatmap and GO enrichment analysis of genes positively and negatively co-expressed with three hub genes (*SPP1*, *MMP2*, and *VCAM1*) in the TCGA-LIHC cohort.
